# Supplementary material for: Genetic identification and hybridization in the seagrass genus Halophila (Hydrocharitaceae) in Sri Lankan waters
Source: PeerJ. 2020 Sep 30;8:e10027. doi: 10.7717/peerj.10027 (PMC7532775; doi:10.7717/peerj.10027)
Supplement: Table S1 [file peerj-08-10027-s003.docx]

Table S.1 List of the reference sequences of *Halophila* included in the molecular analysis done in this study.

| Taxa | Geographic source | Citation | GenBank accession No. of ITS | GenBank accession No. of rcbL |
| --- | --- | --- | --- | --- |
| *Halophila spinulosa* | Australia | Waycott et al. (2002) | AF366439 |  |
| *Halophila*  *tricostata* | Australia | Waycott et al. (2002) | AF366439 |  |
| *Halophila beccarii* | Viet Nam  India | Waycott et al. (2002)  Lucas et al. 2012 | AF366441 | JN225339 |
| *Halophila engelmannii* | USA, Florida | Waycott et al. (2002)  Les et al. 1997 | AF366404 | U80699 |
| *Halophila*  *australis* | Australia | Uchimura et al. (2008) | AB436923 |  |
| *Halophila stipulacea* | Egypt  India  United Arab Emirates  Greece  Italy | Nguyen et al. 2018  Ruggiero and Procaccini 2004  Nguyen et al. 2015 | MF371465  MF371466  AY352613  AY352635  KM609944  MF371467  AY352634  AY352612 | JN225356 |
| *Halophila*  *decipens* | Viet Nam | Nguyen et al. 2013 |  | JX457598 |
| *Halophila nipponica* | Japan, Okinawa  South Korea | Uchimura et al. (2008) Kim et al. 2017 | AB436936  AB436924  KX668189 |  |
| *Halophila*  *johnsonii* | USA, Florida | Waycott et al. (2002) | AF366425 |  |
| *Halophila*  *hawaiiana* | USA, Hawaii | Uchimura et al. (2008) | AB436925 |  |
| *Halophila ovalis* | Okinawa | Uchimura et al. (2005) | AB243970 | JX457593  JX306065 |
| *Halophila minor* | Thailand | Kim et al. 2017  Lucas et al. 2012 | KX668191 | JN225347 |
| *Halophila ovata* | India | Nguyen et al. 2015 | KM609940 |  |
| *Halophila major* | Thailand  Japan, Kagoshima | Nguyen et al. 2014  Tuntiprapas et al. 2015  Uchimura et al. (2008)  Nguyen et al. 2013 | KF620348  KP408265  AB436929 | JX457595 |
